# The influence of $\{10\bar{1}2\}$ twinning on the corrosion behavior of AZ31B magnesium alloy

**DOI:** 10.1016/j.isci.2024.110688

**Published:** 2024-08-13

**Authors:** Xiangyu Li, Baoji Ma, Bin Liu, Jinkui Cao, Liangliang Li, Zhaopeng Xu

**Affiliations:** 1 School of Mechatronic Engineering, Xi’an Technological University, No.2 Xuefuzhonglu Road, Xi’an 710021, China; 2 School of Mechanical and Electrical Engineering, Tongchuan Vocation and Technical College, No.8 Chaoyang Road, Tongchuan 727000, China

**Keywords:** Materials science, Materials chemistry

## Abstract

This study investigates the effect of twinning on the corrosion behavior of AZ31B magnesium alloy using solid solution heat treatment (SHT) and laser shock peening (LSP) techniques. The corrosion characteristics are assessed by scanning electron microscopy (SEM), scanning Kelvin probe force microscopy (SKPFM), zero resistance ammeter (ZRA), scanning vibrating electrode technique (SVET), and electrochemical tests. Results indicate that the twinning region in AZ31B magnesium alloy, enriched with $\{10\bar{1}2\}$ tensile twins induced by laser shock, demonstrates increased corrosion susceptibility. This region exhibits higher electrochemical activity and an accelerated corrosion rate compared to the matrix region. Micro-galvanic coupling between the twinned and matrix regions promotes faster dissolution of the alloy. Additionally, the corrosion product film on the surface is extensively cracked and propagates to the matrix corrosion surface, confirming that $\{10\bar{1}2\}$ tensile twins provide inadequate protection against corrosion in AZ31B alloy.

## Highlights

• A micro-galvanic corrosion occurs between the twin and the matrix
• T-2 samples rich in {$10\bar{1}2$} tension twins exhibit accelerated corrosion as an anode
• The corrosion product film did not provide effective protection
• The order of corrosion tendency is T-2 (0 h) > T-1 (0 h) > T-2 (24 h) > T-1 (24 h)

## Graphical abstract

**Figure:**
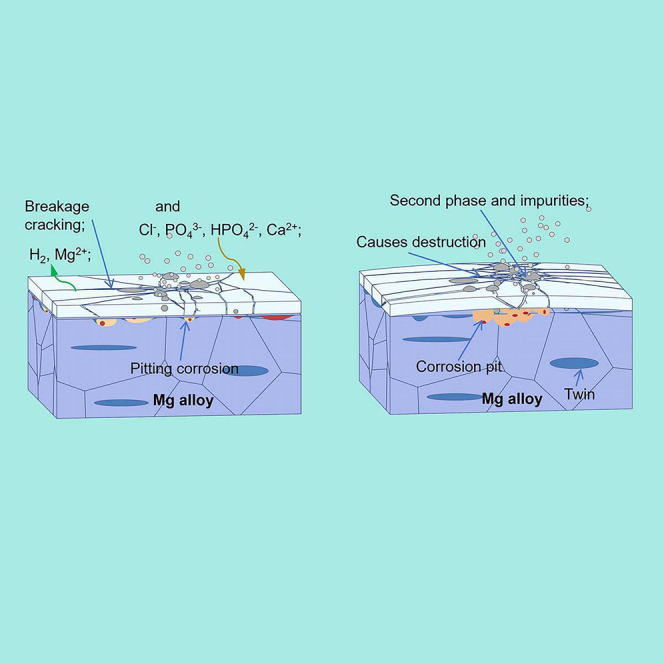

## Introduction

Magnesium alloys are regarded as promising materials in the medical sector due to their favorable biocompatibility and superior mechanical properties, notably their biodegradability, which may mitigate the need for secondary surgeries.^1^^,^^2^^,^^3^^,^^4^^,^^5^^,^^6^^,^^7^ Although AZ31B magnesium alloy is biodegradable, its degradation rate surpasses the bone regeneration rate, potentially compromising the implant’s stability and durability in the body.^8^^,^^9^^,^^10^^,^^11^^,^^12^ These disparities impose limitations on its clinical applications. Currently, enhancing the corrosion resistance of magnesium alloys through deformation presents a viable approach for their use in medical implants. Nonetheless, the hexagonal close-packed (HCP) structure of magnesium alloys features limited slip systems, resulting in poor formability and ductility. Additionally, significant twinning occurs during deformation, causing abrupt changes in crystal orientations within the parent grain when twins are activated.^13^^,^^14^^,^^15^ This phenomenon, a type of lattice distortion, not only substantially affects the mechanical attributes of the metal but may also influence the corrosion resistance of the magnesium alloy matrix.^16^^,^^17^^,^^18^

According to the study by Gerashi et al.,^19^ the effects of twinning and crystal structure on the corrosion behavior of magnesium alloys are explored in detail. This research elucidates how these factors influence the corrosion process, including the corrosion rate and the formation of corrosion products, and offers guidance for enhancing the corrosion resistance of magnesium alloys. Xiong et al.^20^ introduced twins of varying densities into ZK60 magnesium alloy under different stress conditions. Electrochemical tests revealed that the corrosion rates of the alloys increased in the following order: ED-9% (high twin density) < original sample < ED-3% (low twin density). This variation correlates with the degree of microstructural deformation; in the ED-3% samples, $\{10\bar{1}2\}$ tensile twins were only observed in larger grains,^21^ resulting in orientation disparities between twinned and twin-free zones, which induced galvanic corrosion.^22^ The optimal twin density to improve corrosion resistance has also been confirmed by Sabbaghian et al.^18^ Mg-4Zn alloys subjected to extrusion deformation showed higher twin volume fractions in the TD and 45° samples. Characterization by EBSD of the 45° samples demonstrated that the basal faces had higher corrosion resistance compared to the pyramidal and prismatic faces, effectively reducing the corrosion rate. Moreover, the 45° samples exhibited higher charge transfer resistance (R) and film resistance (R), indicating superior resistance to corrosive media. Zou et al.^17^ suggested that twinning in Mg-Y alloys might provide some protection, acting as micro-anodes where a film preferentially forms on the twin boundary faces, thus stabilizing the alloy’s surface film and enhancing its corrosion resistance in corrosive environments. This implies that the twin structure might play a role in mitigating corrosion.

Although existing studies indicate that twinning may enhance the corrosion resistance of magnesium alloys, other research suggests that the twin regions, due to micro-galvanic corrosion between the twins and the matrix, could become susceptible areas, accelerating the overall corrosion rate of these alloys.^17^^,^^23^ These conflicting perspectives highlight the complex role of twins in influencing the corrosion behavior of magnesium alloys. For instance, Zhou et al.^24^ observed that intergranular corrosion was predominant in AZ31-H24 magnesium alloy in a 3.5 wt.% NaCl solution, proposing that twins may exacerbate this type of corrosion and decrease the alloy’s corrosion resistance. This suggests that changes in twin structure could significantly impact corrosion behavior. Similarly, in studies by Schmutz et al.^25^ on a large-grained magnesium sample with defined defect structures, corrosion was noted to propagate at twin boundaries, indicating a critical interaction at these sites. Optimizing twin density is essential for achieving the best corrosion resistance. Moreover, research by Song et al. and Pawar et al.^26^^,^^27^ has underscored the importance of understanding the relationship between twinning and corrosion. Their findings suggest that the basal plane {0002} of magnesium alloys exhibits superior corrosion resistance compared to the prismatic plane $\{10\bar{1}0\}$. It appears that mild galvanic corrosion between twins and the matrix can accelerate the dissolution of the magnesium matrix, while also potentially facilitating the formation of a protective surface film. Thus, twinning may play various roles in the corrosion behavior of magnesium alloys. Although these effects have been documented, the precise mechanisms underlying these observations remain unclear. Gerashi et al.^19^ have elaborated on the roles of twinning and texture in the corrosion processes of magnesium alloys, yet these discussions often focus only on specific aspects of twinning.

Based on the preceding summary, three key questions emerge: (1) what is the impact of twinning on the corrosion resistance of AZ31B magnesium alloy? (2) Do twins promote the formation of a surface film? (3) If so, is the surface film on AZ31B magnesium alloy protective? Addressing these questions is crucial for understanding the role of twinning in the corrosion processes of magnesium alloys and for enhancing their corrosion performance. In this study, as-cast AZ31B magnesium alloy plates are initially subjected to uniform heat treatment and laser shock peening to produce samples rich in $\{10\bar{1}2\}$ twins. These samples are then tested in 37 ± 0.1°C simulated body fluid to evaluate the effect of twinning on the corrosion behavior of the AZ31B magnesium alloy matrix.

### Materials and methods

#### Preparation and treatment of materials

The study utilizes as-cast AZ31B magnesium alloy samples to minimize the impact of original tissue texture on corrosion. The chemical composition of the samples conforms to the national standard GBT5153-2003, as detailed in Table 1. Each sample measures 10 × 10 × 5 mm^3^ and undergoes uniform heat treatment in a vacuum atmosphere tube furnace. The sample after heat treatment is labeled as T-1, whereas the sample subjected to both laser shock and uniform heat treatment is labeled as T-2. For T-1, the treatment temperature is set at 520°C for 4 h, followed by cooling to room temperature within the furnace. The treatment parameters for T-2 include a laser power of 4.95 GW/cm^2^, two impacts, a 50% spot overlap rate, and a subsequent annealing at 250°C for 1 h to alleviate the residual stress induced by the laser shock. In this study, T-1 refers to the uniformly heat-treated sample without twins, and T-2 refers to the sample containing twins after laser shock. The residual stress in the T-2 sample, found to be insignificant upon detection, is not discussed further in this paper.

**Table 1:** Composition ratio of AZ31B magnesium alloy

Element	Al	Zn	Mn	Si	Ca	Cu	Ni	Fe	Mg
Percent (wt.%)	3.2	1.4	0.7	0.07	0.04	0.01	0.001	0.003	Bal

#### Microstructure analysis

The surface of the impact sample was progressively polished using metallographic sandpaper ranging from #1,000 to #5,000, followed by further mirror polishing with W1.0 diamond grinding paste. After polishing, the sample was ultrasonically cleaned in an ethanol (CH_3_CH_2_OH) solution. Metallographic etching was conducted using a solution composed of 10 mL nitric acid, 70 mL acetic acid, 10 mL distilled water, and 4.2 g picric acid, with an etching duration of 5–15 s. Subsequently, the sample was immersed in simulated body fluid at 37 ± 0.1°C, the composition of which is detailed in Table 2. To evaluate the corrosion morphology, corrosion products on the sample surface were removed following immersion, and the sample was then soaked in a chromic acid solution (200 g/L CrO_3_ + 10 g/L AgNO_3_) for 5 min. The microstructure was characterized using a Nikon LV100ND metallographic optical microscope (OM), a Zeiss laser confocal microscope (Smartproof 5), a Zeiss EVO scanning electron microscope (SEM), and a Zeiss electron backscatter diffraction microscope.

**Table 2:** Major components of the simulated body fluids

Component	Na^+^	K^+^	Mg^2+^	Ca^2+^	Cl^−^	HCO_3_^−^	HPO_4_^2−^	SO_4_^2−^
Percent (mM/L)	142.0	5.0	1.5	2.5	103.0	10.0	1.0	0.5

#### Scanning Kelvin probe force microscope

The Bruker Nano Inc. SKPFM is utilized to measure the Volta potential difference between the matrix and twins of the T-2 sample in the working function mode. SKPFM is a technique designed for assessing surface potentials at the nanoscale. When the AFM probe contacts the sample surface, charge transfer induces a potential on the surface. By quantifying the potential difference between the probe and the sample, the local potential distribution on the sample surface is determined.

#### Coupling test

To compare the corrosion behavior of AZ31B magnesium alloy samples T-1 and T-2, two distinct techniques, ZRA Test and SVET Test, are employed to assess the galvanic corrosion following coupling. Both tests are conducted in a 37 ± 0.1°C simulated body fluid solution. The ZRA test utilizes the CHI600E electrochemical test system. For this test, T-1 and T-2 samples are each connected to a wire, encapsulated in epoxy resin to ensure fixation and sealing, with an exposed detection area polished to 10 mm^2^. The T-1 sample is connected to the ground of the electrochemical workstation, whereas the T-2 sample is attached to the electrode end. The workstation records the current every 5 s over a total duration of 2 h.

The SVET test is performed using the VersaScan micro-area scanning electrochemical workstation. The T-1 and T-2 samples are coupled using conductive silver paste and connected via wires, followed by encapsulation in epoxy resin. The detection surface is polished to a mirror finish exposing an area of 200 mm^2^. The SVET tests are conducted over 1 and 2 h in a 37 ± 0.1°C simulated body fluid environment. During the test, the probe tip is maintained at a distance of 100 μm from the sample, utilizing a Pt-Ir microelectrode with a tip diameter of 16 μm and an amplitude of 32 μm.

#### Electrochemical experiment

The PARSTAT electrochemical workstation is employed to perform electrochemical measurements in 37 ± 0.1°C simulated body fluid. Measurements utilize a three-electrode sealed electrochemical cell, incorporating a saturated calomel electrode (SCE) as the reference electrode and a platinum electrode as the auxiliary electrode. The electrochemical corrosion sample is connected to a copper wire, encapsulated in epoxy resin, and the detection area, polished to 100 mm^2^, is exposed. The dynamic potential polarization curve test is conducted with a scanning speed of 1.0 mV/s. The anode and cathode scans are carried out separately to mitigate the effects of hydrogen evolution on the sample during corrosion. Electrochemical impedance spectroscopy is performed over a frequency range from 0.1 Hz to 100 kHz, with an excitation signal consisting of a sine wave of 5 mV amplitude, and data analysis is facilitated using ZSimpWin software. To ensure the stability of the electrolyte and to eliminate initial interference, a delay of 10 min is introduced before conducting the experiments. All electrochemical tests are replicated 2–3 times following the same procedure to confirm the reliability of the results.

## Results and discussion

### Microstructure characterization

**Figure 1:**
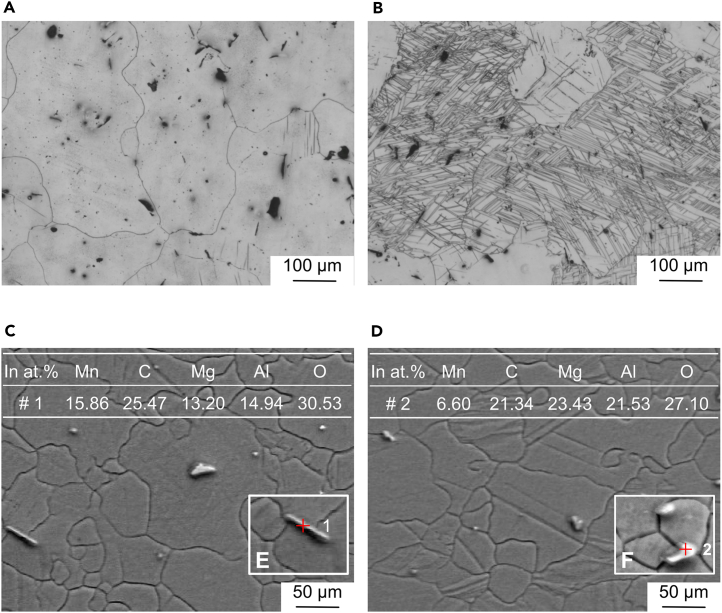
Micrographs of the surface of the AZ31B magnesium alloy sample (A and B) are metallographic images of the surface of the T-1 and T-2 samples after etching; (C and D) are BSE-SEM micrographs of the surface of the T-1 and T-2 samples. The most significant difference between them in microstructure is the presence of twin structure in T-2 specimen.

**Figure 2:**
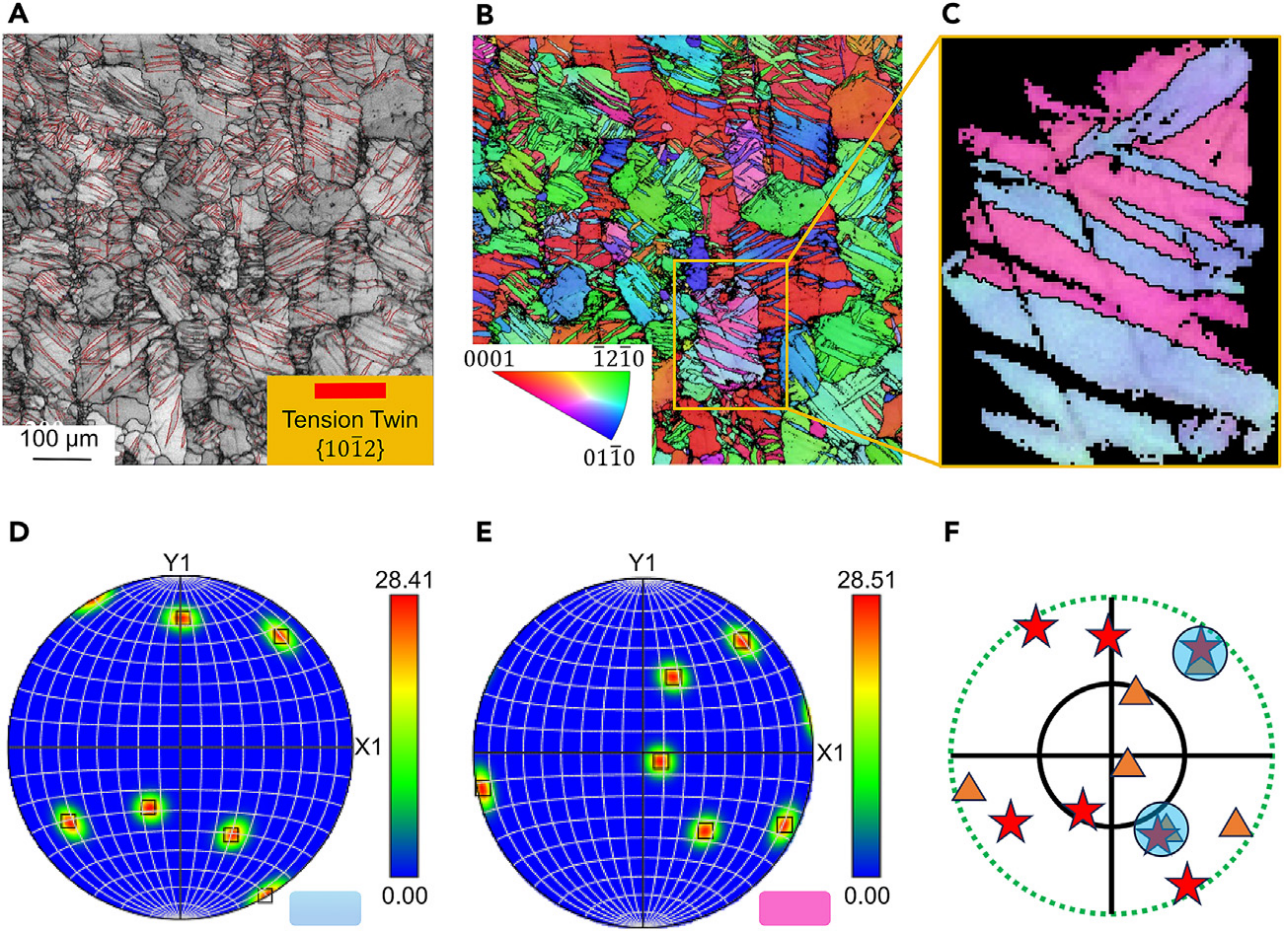
EBSD analysis of the surface of AZ31B magnesium alloy sample T-2 (A), (B), and (C) are inverse pole figures; (D) and (E) are pole figures; (F) is the corresponding (0002) pole figure. It can be clearly observed that a large number of twins are successfully induced on the pre-etched surface of T-2 specimen, which are of type $\{10\bar{1}2\}$ tensile twins. It is observed that the samples have strong base texture from the inverse polar diagram and the inverse polar diagram.

Figures 1A and 1B display metallographic microscope images of the as-cast AZ31B magnesium alloy samples. The images illustrate that the sample T-2 (Figure 1B) contains a significantly higher number of twin structures compared to sample T-1 (Figure 1A). The BSE SEM micrographs in Figures 1C and 1D reveal that both samples T-1 and T-2 exhibit point-like and strip-like secondary phases, primarily composed of Mg, Al, and Mn elements. Given the minor proportion of these phases, this study does not focus on the micro-galvanic corrosion between the secondary phases and the Mg matrix. The grain size of samples T-1 and T-2 is approximately 100 μm. The most notable distinction in their microstructures is the presence of twin structures in sample T-2. Prior to corrosion exposure, sample T-2 underwent EBSD testing, as depicted in Figure 2. This analysis confirmed the successful induction of a substantial number of {$10\bar{1}2$} tensile twins on the pre-corrosion surface of sample T-2. Additionally, the inverse pole figure reveals a strong basal texture in the sample.

### SKPFM analysis

**Figure 3:**
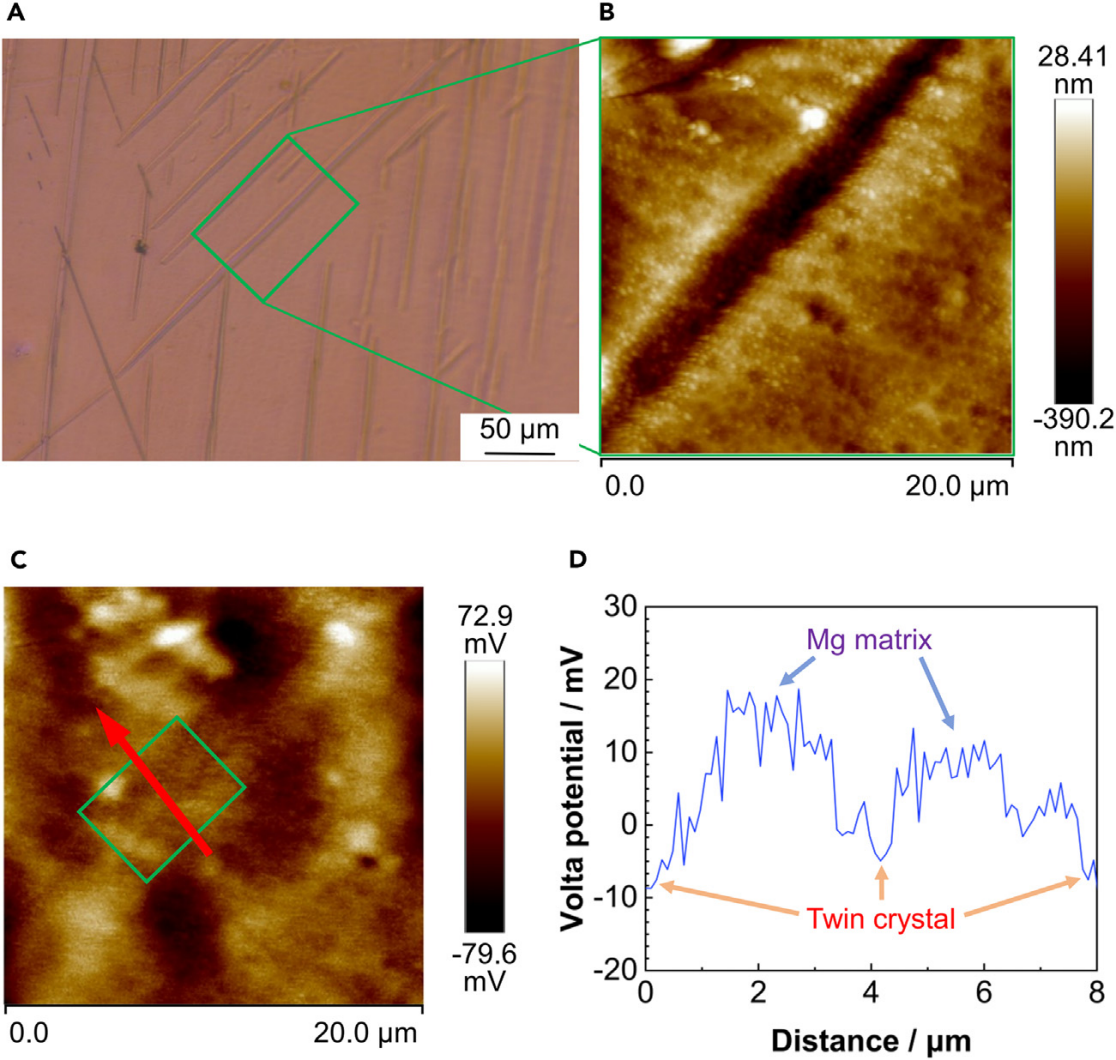
SKPM detection analysis diagram of T-2 sample (A) Optical microscope image of the sample surface. (B) Morphology of $\{10\bar{1}2\}$ tensile twins. (C) Potential distribution map. (D) Line analysis results. This finding indicates that the $\{10\bar{1}2\}$ tensile twin area exhibits higher chemical activity than the magnesium matrix. The minimal potential difference also suggests that micro-galvanic corrosion is likely to occur between $\{10\bar{1}2\}$ tensile twins and the magnesium matrix.

To investigate the chemical activity differences between $\{10\bar{1}2\}$ tensile twins and the matrix in AZ31B magnesium alloy, the T-2 sample was etched using metallographic techniques and then analyzed using SKPFM in air. This process was aimed at obtaining the Volta potential difference between $\{10\bar{1}2\}$ tensile twins and the magnesium matrix to assess their relative activities. Figure 3 presents the SKPFM detection analysis results for the AZ31B magnesium alloy sample. SKPFM scanned the area within the square shown in Figure 3A, with the morphological details of this area depicted in Figure 3B. Figure 3D illustrates the potential line scan analysis at the arrow indicated in the potential distribution map in Figure 3C. According to the Volta potential mapping, the magnesium matrix exhibits a higher potential relative to the twin plane within the twin area; the potential difference between them is approximately 30 mV. Areas with lower potential, such as $\{10\bar{1}2\}$ tensile twins, are more prone to act as anodes. This finding indicates that the $\{10\bar{1}2\}$ tensile twin area exhibits higher chemical activity than the magnesium matrix. The minimal potential difference also suggests that micro-galvanic corrosion is likely to occur between $\{10\bar{1}2\}$ tensile twins and the magnesium matrix. It is hypothesized that $\{10\bar{1}2\}$ tensile twins may corrode preferentially in a corrosive solution.

### Corrosion morphology observation

**Figure 4:**
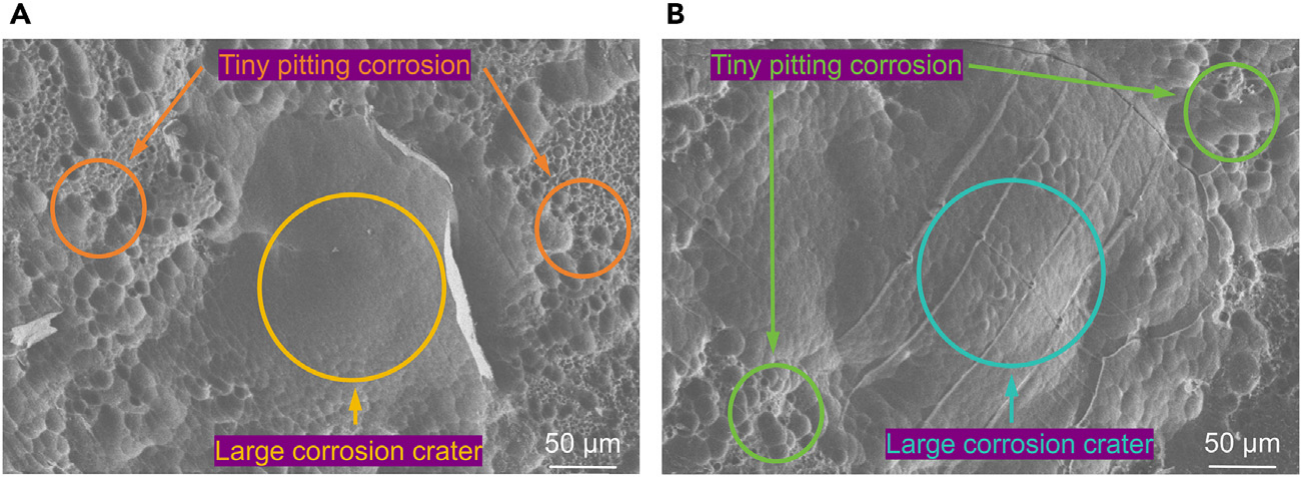
The surface morphology after soaking in 37 ± 0.1°C simulated body fluid for 12 h and removing corrosion products (A) T-1 sample. (B) T-2 sample. Figure 4B highlights the presence of twin structures within the larger corrosion pits of sample T-2, in stark contrast to sample T-1 shown in Figure 4A. This disparity suggests that micro-galvanic corrosion has occurred between the twins and the magnesium matrix.

The AZ31B magnesium alloy samples T-1 and T-2 were polished before undergoing corrosion soaking in 37 ± 0.1°C simulated body fluid for 12 h, after which the corrosion products were removed. The resultant surface morphology, depicted in Figure 4, reveals uneven corrosion characteristics, with both samples exhibiting smaller pitting pits alongside larger corrosion pits. Figure 4B highlights the presence of twin structures within the larger corrosion pits of sample T-2, in stark contrast to sample T-1 shown in Figure 4A. This disparity suggests that micro-galvanic corrosion has occurred between the twins and the magnesium matrix.

**Figure 5:**
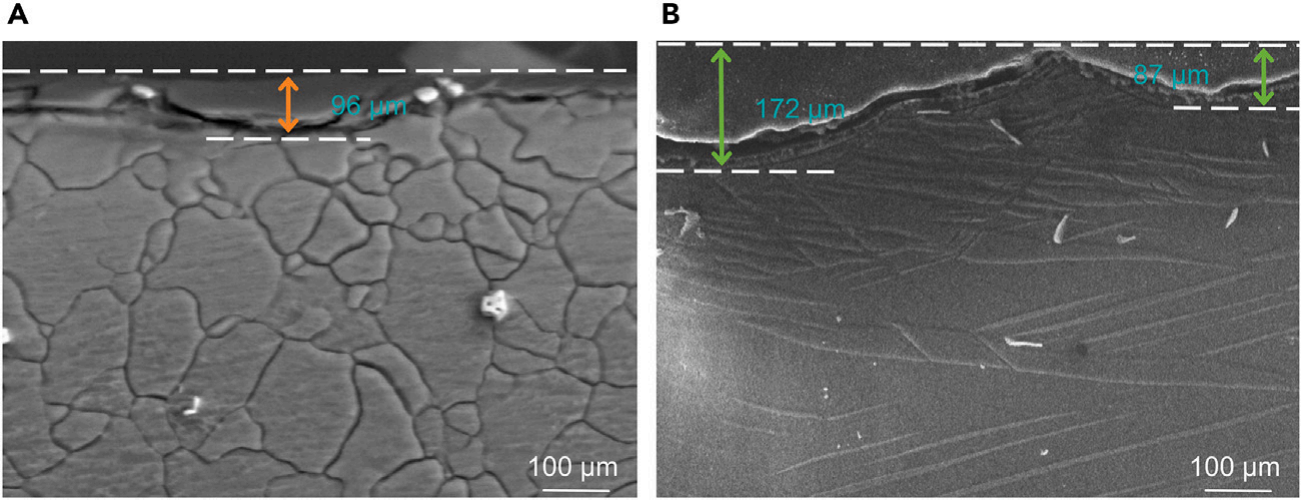
Cross-sectional morphology after soaking in 37 ± 0.1°C simulated body fluid for 12 h and removing corrosion products (A) T-1 sample. (B) T-2 sample. This observation confirms that regions with twins are more susceptible to corrosion compared to the Mg matrix, aligning with the SKPFM analysis results shown in Figure 3.

Figure 5 presents SEM images of the cross-sections of samples T-1 and T-2 after 12 h of corrosion soaking. The depth of the corrosion pit in the twin-free T-1 sample measures 96 μm, whereas the pit in the twin-containing T-2 sample is deeper at 172 μm, with the twins clearly visible in Figure 5B. This observation confirms that regions with twins are more susceptible to corrosion compared to the Mg matrix, aligning with the SKPFM analysis results shown in Figure 3.

**Figure 6:**
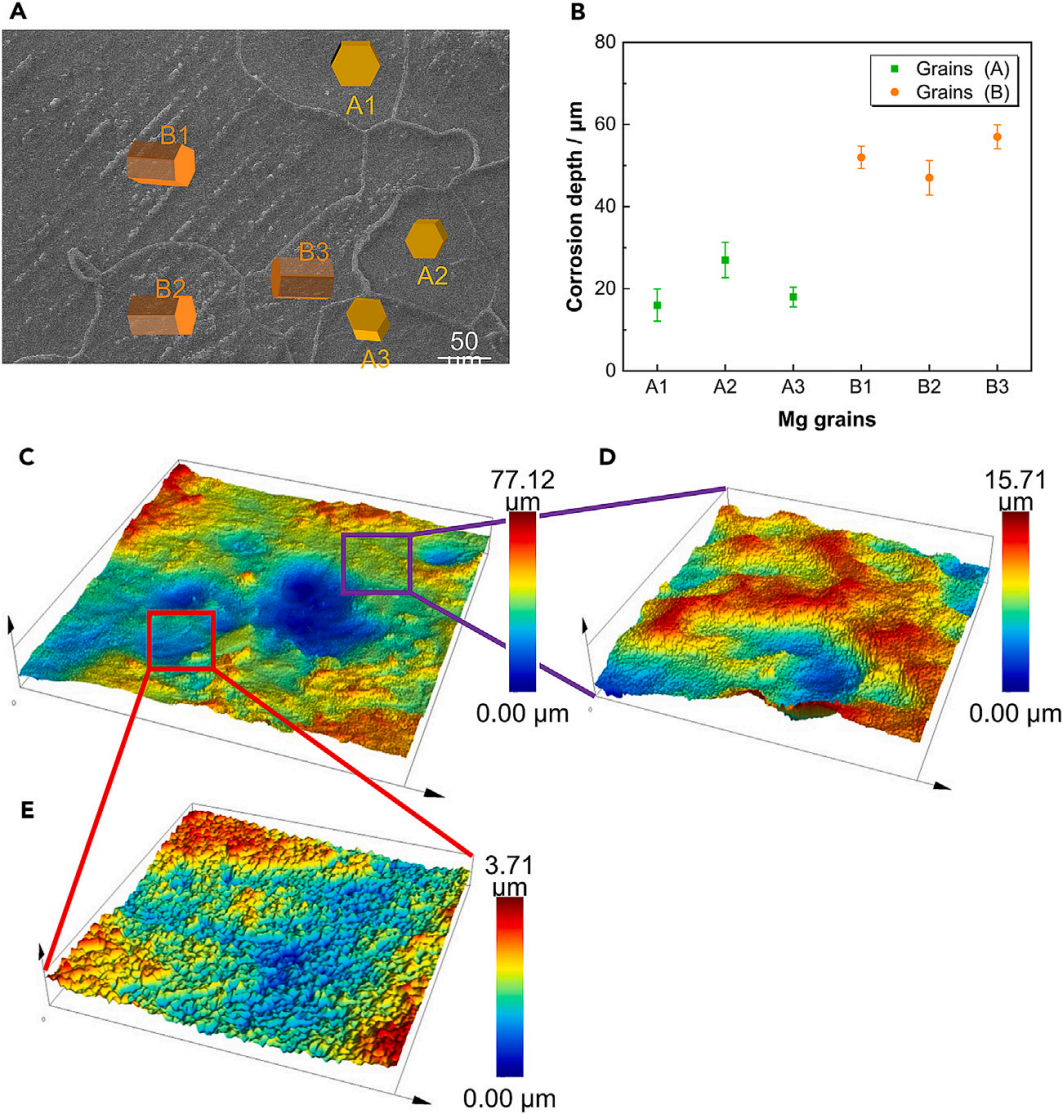
Morphology characterization of corrosion products after removal (A) Characterization of the corrosion surface after corrosion soaking for 2 h and removing corrosion products. (B) The chart shows the average corrosion depth of each Mg grain. (C–E) The change in surface corrosion depth after removing corrosion products. (please note the different color scales). The average corrosion depth of Grains A (A1–A3) is 20.33 μm, significantly less than that of Grains B (B1–B3), which measures 52 μm, indicating that grains with non-basal orientations exhibit lower corrosion resistance.

**Figure 7:**
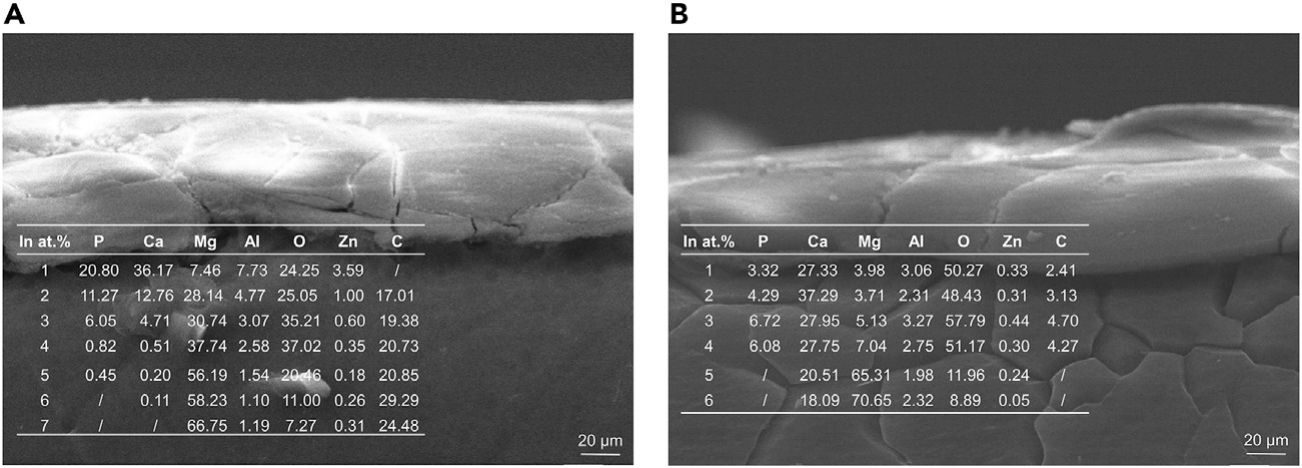
Cross-sectional morphology after soaking in 37 ± 0.1°C simulated body fluid for 24 h (A) T-1 sample. (B) T-2 sample. Analysis of the elemental distribution within the corrosion product films reveals similar compositions for both samples, consisting predominantly of P, Ca, Mg, Al, O, Zn, CP, and Ca. The concentrations of P and Ca are slightly higher in the film of sample T-1 than in T-2, suggesting that elements such as P and Ca precipitate from the simulated body fluid onto the alloy surface, forming a Ca-P compound.

Furthermore, after soaking in 37 ± 0.1°C simulated body fluid for 2 h, the backscattered electron image of the corrosion surface for sample T-2 is shown in Figure 6A. Grains with basal crystal orientation are labeled Grains A (A1–A3), whereas those with non-basal orientation are labeled Grains B (B1–B3). As depicted in Figure 6B, Grains A (A1–A3) and Grains B (B1–B3) exhibit different corrosion depths. The average corrosion depth of Grains A (A1–A3) is 20.33 μm, significantly less than that of Grains B (B1–B3), which measures 52 μm, indicating that grains with non-basal orientations exhibit lower corrosion resistance. The laser confocal microscope analysis, shown in Figure 7, further illustrates that adjacent grains experience varying corrosion depths. Figures 6D and 6E demonstrate notable differences in the surface contours of the two grain types. This variability suggests that the AZ31B magnesium alloy experiences differing corrosion depths during the corrosion process in simulated body fluid, contingent on the crystal orientations of the grains.

### Lateral morphology of the surface film

After soaking in 37 ± 0.1°C simulated body fluid for 24 h, the cross-sectional morphology of the corrosion product films on samples T-1 and T-2 is depicted in Figure 7. The films on both samples exhibit a dense and uniform surface; however, they also display characteristics of looseness and cracking. These cracks extend directly into the matrix, with the surface film of sample T-1 being slightly thicker than that of sample T-2. Analysis of the elemental distribution within the corrosion product films reveals similar compositions for both samples, consisting predominantly of P, Ca, Mg, Al, O, Zn, CP, and Ca. The concentrations of P and Ca are slightly higher in the film of sample T-1 than in T-2, suggesting that elements such as P and Ca precipitate from the simulated body fluid onto the alloy surface, forming a Ca-P compound. The Ca-P compound film is dense and resistant to degradation by Cl^−^ ions.^28^ Consequently, the cross-sectional morphology analysis suggests that the corrosion product film of sample T-1 is thicker, inferring a relatively higher protective quality compared to sample T-2.

**Figure 8:**
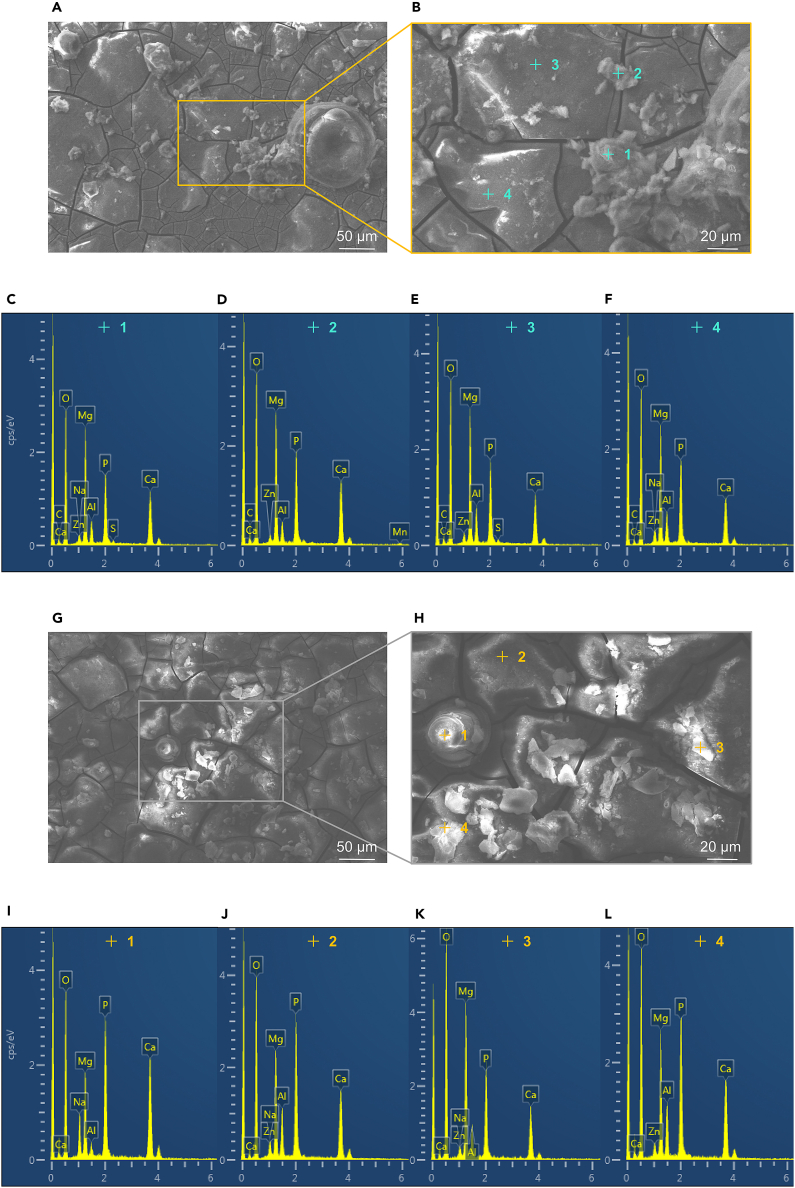
Surface morphology after soaking in 37 ± 0.1°C simulated body fluid for 24 h (A) Surface morphology of T-1 sample. (B) Partial area of (A). (C–F) EDS test results of T-1 sample. (G) Surface morphology of T-2 sample. (H) Partial area of (G). (I–L) EDS test results of T-2 sample. As the soaking duration increases, the atomic percentage of elements C, O, and P in the EDS spectra rises, whereas that of Mg decreases, suggesting the presence of Mg(OH)_2_, Ca-Mg, and Mg_3_(PO4)_2_ in the corrosion film of the AZ31B magnesium alloy.

After a 24-h soak in simulated body fluid, the surface morphology of the corrosion product films on the T-1 and T-2 samples is depicted in Figure 8. Both samples exhibit relatively flat corrosion product films, with the surfaces of the corrosion pits covered by gray corrosion products. These products consist of the film itself and aggregated particles on its surface. Notably, the films on both T-1 and T-2 samples display cracks and segmentation into small pieces. The chemical composition of the corrosion product films was analyzed using energy dispersive spectroscopy (EDS), with results for marked areas in Figures 8A and 8G presented in Figures 8C–8F and 8I–8L, respectively. A comparison of the EDS results between the T-1 and T-2 samples reveals that the phase of the corrosion products of the AZ31B magnesium alloy remains unchanged, predominantly comprising Mg(OH)_2_. The corrosion product film on the T-1 sample is relatively smoother than that on the T-2 sample. As the soaking duration increases, the atomic percentage of elements C, O, and P in the EDS spectra rises, whereas that of Mg decreases, suggesting the presence of Mg(OH)_2_, Ca-Mg, and Mg_3_(PO_4_)_2_ in the corrosion film of the AZ31B magnesium alloy.

**Figure 9:**
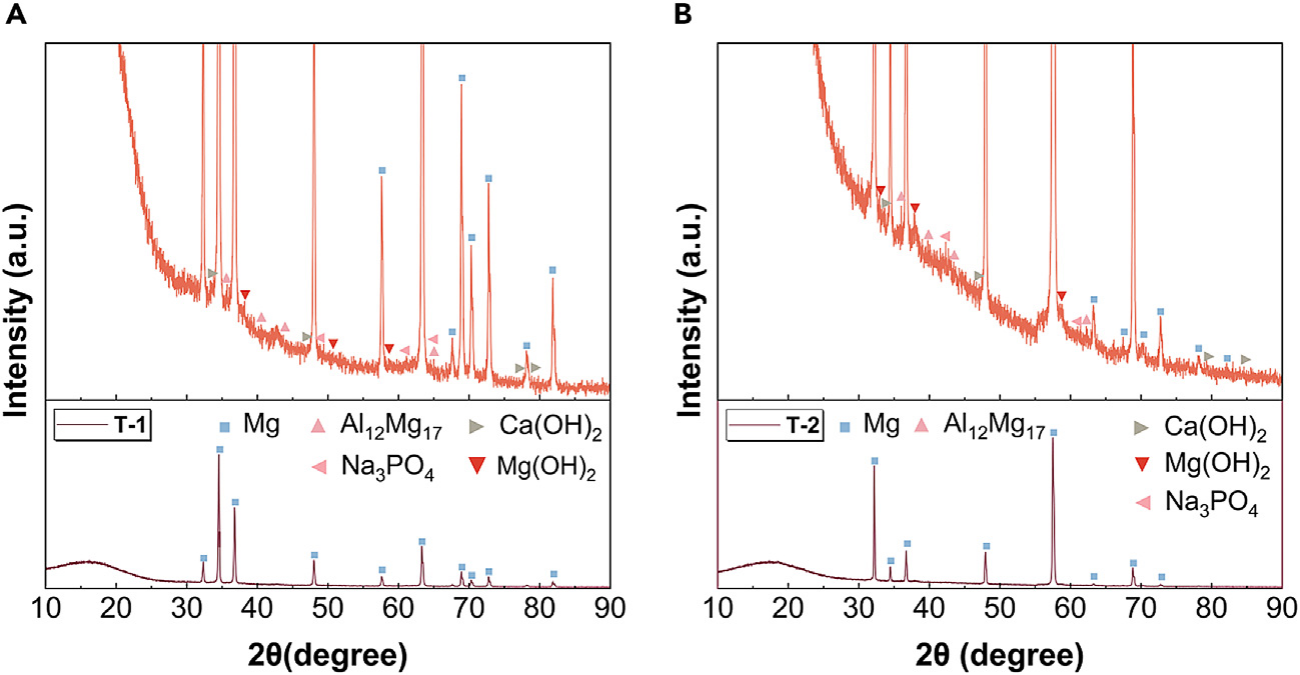
XRD test results after soaking in 37 ± 0.1°C simulated body fluid for 24 h (A) T-1 sample. (B) T-2 sample. This analysis confirms that elements in the simulated body fluid interact with the alloy surface during corrosion, forming compounds that may influence the degradation rate of the AZ31B magnesium alloy.

The X-ray diffraction (XRD) analysis of the corrosion product films on T-1 and T-2 samples is presented in Figure 9. Analysis of the XRD peaks reveals the crystal plane indices and corresponding peak values for the T-1 and T-2 samples. For the T-1 sample, the peak values and indices for magnesium are as follows: 32.33°-(100), 34.55°-(002), 36.77°-(101), 48.01°-(102), and 57.59°-(110). The peaks for aluminum-magnesium alloy (Al_12_Mg_17_) are at 35.67°-(411), 40.14°-(332), 43.64°-(510), and 64.94°-(721). The peaks for magnesium hydroxide (Mg(OH)_2_) occur at 38.14°-(101), 50.74°-(102), and 58.56°-(110) and those for calcium hydroxide (Ca(OH)_2_) are located at 33.76°-(101), 47.14°-(102), 77.80°-(004), and 78.87°-(113). Sodium phosphate (Na_3_PO_4_) shows peaks at 48.89°-(400), 61.13°-(422), and 64.94°-(511). For the T-2 sample, the magnesium peaks are at 32.16°-(100), 34.45°-(002), 36.72°-(101), 47.96°-(102), and 57.49°-(110). The Al_12_Mg_17_ alloy shows peaks at 36.02°-(411), 40.08°-(332), 43.88°-(510), and 62.24°-(710). Magnesium hydroxide peaks are at 32.94°-(100), 37.82°-(101), and 58.41°-(110), and calcium hydroxide at 33.66°-(101), 46.63°-(102), 78.95°-(113), and 84.58°-(211). Sodium phosphate is identified at 42.31°-(222) and 60.93°-(420).

This analysis confirms that elements in the simulated body fluid interact with the alloy surface during corrosion, forming compounds that may influence the degradation rate of the AZ31B magnesium alloy. Further investigation into how these interactions affect the alloy’s degradation in simulated body fluid is warranted.

### Coupling test

**Figure 10:**
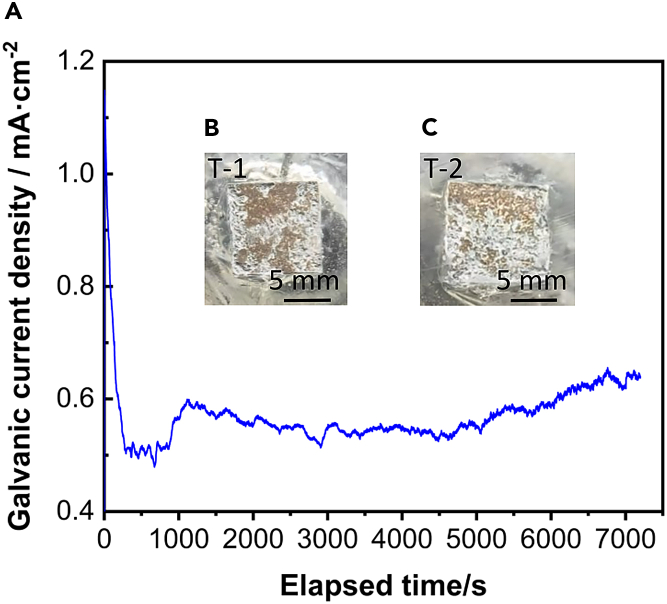
Zero resistance ammeter test (A) Galvanic current density in simulated body fluid after coupling of T-1 and T-2 samples. (B and C) are the morphologies of samples T-1 and T-2, respectively. The results indicated that the current density curve consistently exhibited positive values, reaching a minimum of 0.48 mA cm^−2^ at 674 s, suggesting that the T-2 sample consistently acted as an anode undergoing dissolution.

To elucidate the corrosion behavior of alloys with and without twinning, T-1 and T-2 samples were coupled and subjected to soaking. The corrosion behavior of these samples was subsequently analyzed using a zero resistance ammeter test, as depicted in Figure 10. The results indicated that the current density curve consistently exhibited positive values, reaching a minimum of 0.48 mA cm^−2^ at 674 s, suggesting that the T-2 sample consistently acted as an anode undergoing dissolution. As shown in Figure 10C, extensive corrosion was evident on the surface of the T-2 sample, whereas the T-1 sample exhibited relatively minor localized corrosion. This observation aligns with the earlier analysis of corrosion morphology, where the current density results for both T-1 and T-2 samples were found to be consistent, indicating a preferential corrosion of the T-2 sample when coupled, serving as micro-anodes.

**Figure 11:**
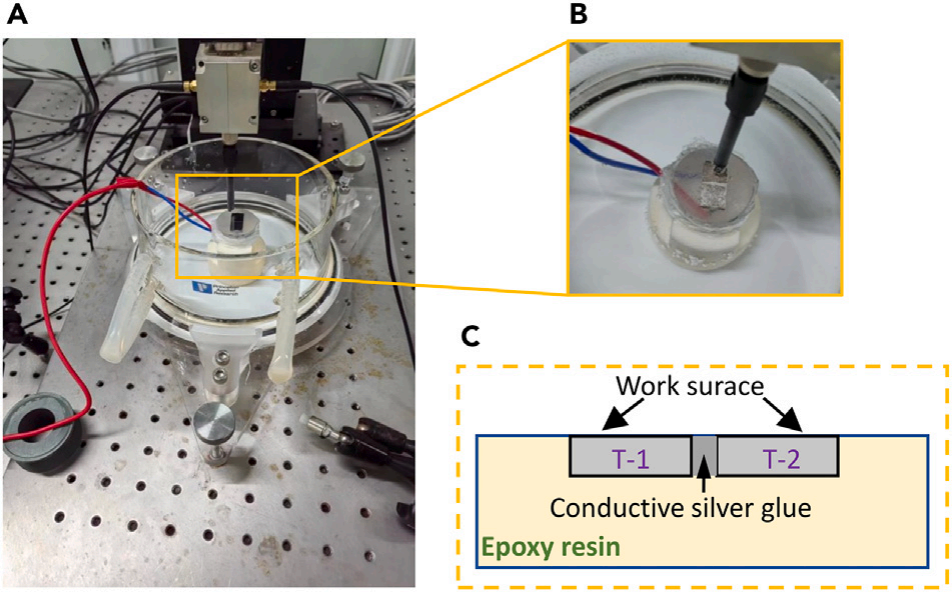
Position diagram of the coupling relationship of T-1 and T-2 samples (A and B) are the actual test drawings, and (C) is the schematic diagram of the sample position relationship.

**Figure 12:**
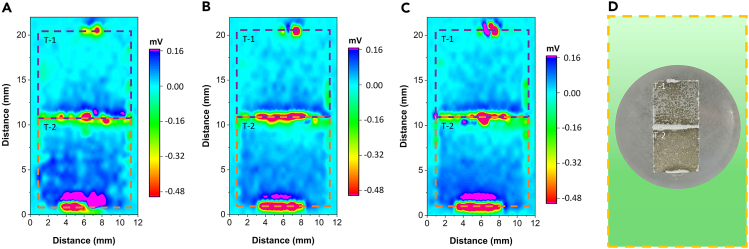
SVET results of T-1 and T-2 samples after coupling in simulated body fluid (A) Soaking for 0 h. (B) Soaking for 1 h. (C) Soaking for 2 h. (D) Macroscopic morphology after soaking for 2 h. The SVET results revealed that during soaking intervals of 0 h, 1 h, and 2 h, the surface voltage distribution of the T-1 sample was predominantly negative, whereas that of the T-2 sample was predominantly positive.

The coupling configuration of the T-1 and T-2 samples is illustrated in Figure 11, with the SVET results presented in Figure 12. An anomalous red area in Figure 12, attributed to the conductive silver glue used during the coupling of the samples, is noted but not discussed further here. The SVET results revealed that during soaking intervals of 0 h, 1 h, and 2 h, the surface voltage distribution of the T-1 sample was predominantly negative, whereas that of the T-2 sample was predominantly positive. This trend in voltage distribution, consistent across different soaking times, shows that the surface current distribution of the T-1 sample was mainly negative (cathodic), whereas that of the T-2 sample was primarily positive (anodic). These findings corroborate the ZRA test results. Furthermore, macroscopic morphological analysis after soaking, as shown in Figure 12C, indicates that the corrosion severity on the surface of the T-1 sample was milder compared to the T-2 sample, which exhibited more severe corrosion due to the presence of a large number of {$10\bar{1}2$} tensile twins.

### Electrochemical experiment

**Figure 13:**
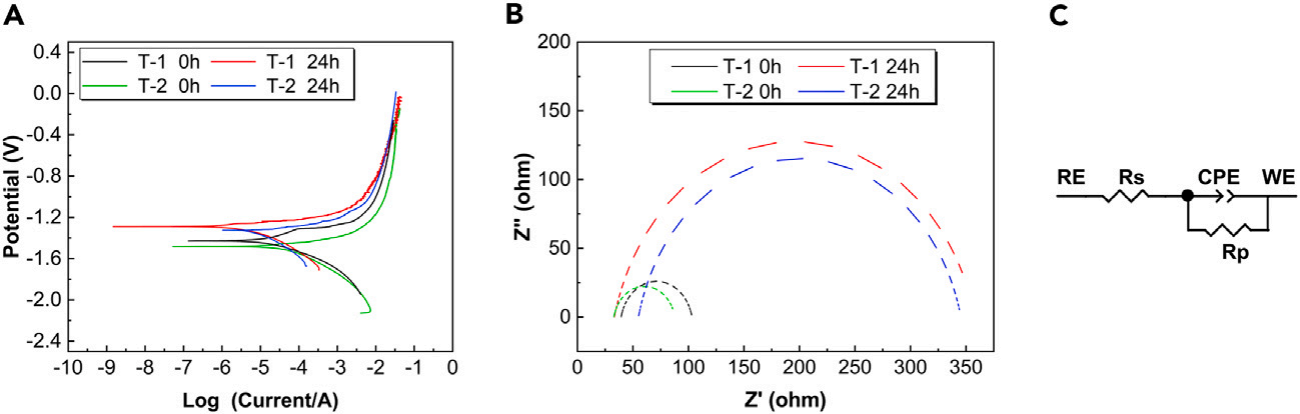
Electrochemical test results of T-1 and T-2 samples (A) Tafel curve. (B) Nyquist diagram. (C) Equivalent circuit diagram. The T-1 sample exhibits a lower corrosion current, suggesting better corrosion resistance compared to the T-2 sample, which contains a high number of $\{10\bar{1}2\}$ tensile twins and demonstrates faster corrosion and reduced resistance. The differences in the impedance spectrum curves of T-1 and T-2 samples may stem from variations in corrosion rates, influencing the structure of the MgO/Mg(OH)_2_ oxide layer formed on the sample surfaces.

The AZ31B magnesium alloy samples, T-1 and T-2, were immersed in 37 ± 0.1°C simulated body fluid to conduct dynamic potential tests over 0-h and 24-h durations. The dynamic potential polarization curves are presented in Figure 13A. The Tafel curve fitting results are summarized in Table 3. These results indicate that as the twin density in the AZ31B magnesium alloy samples increases, the corrosion current density also increases, whereas the corrosion potentials remain closely matched. Initially, at 0 h, the corrosion potentials for T-1 and T-2 samples are −1.43 V and −1.47 V, respectively, with corresponding corrosion current densities of 1.20 × 10^−5^ A/cm^2^ and 1.22 × 10^−5^ A/cm^2^. After 24 h, the corrosion current densities are 7.75 × 10^−6^ A/cm^2^ for T-1 and 1.06 × 10^−5^ A/cm^2^ for T-2, indicating that increased twin density leads to higher corrosion rates. The T-1 sample exhibits a lower corrosion current, suggesting better corrosion resistance compared to the T-2 sample, which contains a high number of {$10\bar{1}2$} tensile twins and demonstrates faster corrosion and reduced resistance.

**Table 3:** Fitting results of Tafel curve of T-1 and T-2 samples

Samples	*I*corr (A/cm^2^)	*E*corr (V)
T-1 0h	1.20 × 10^−5^	−1.43
T-1 24h	7.75 × 10^−6^	−1.31
T-2 0h	1.22 × 10^−5^	−1.47
T-2 24h	1.06 × 10^−5^	−1.37

To further analyze the electrochemical corrosion behavior before and after an increase in twin density within a simulated body fluid environment, electrochemical impedance spectroscopy tests were conducted on samples after 0 h and 24 h of soaking. Figure 13B displays the electrochemical impedance spectrum curves of AZ31B magnesium alloy samples T-1 and T-2 post-soaking. The fitting results are detailed in Table 4, where Rs represents the resistance in the electrolyte, Rp denotes the reaction resistance, and CPE1 characterizes a double-layer capacitor with the electrode surface in contact with the solution (constant phase element).

**Table 4:** Fitting results of EIS curve of T-1 and T-2 samples

Samples	Rs (Ω·cm^2^)	R_p_ (Ω·cm^2^)	CPE1-T/(Ω ^−1^ cm^-2^·s^-n^)	CPE1-T
T-1 0h	39.39	63.8	1.71 × 10^−5^	0.87133
T-1 24h	33.22	324.9	3.24 × 10^-^5	0.8478
T-2 0h	32.85	54.93	1.77 × 10^−5^	0.87067
T-2 24h	54.94	290.5	3.00 × 10^−5^	0.8543

Nyquist diagrams show that the impedance spectra of both T-1 and T-2 samples are consistent both immediately after immersion and 24 h later, with high-frequency capacitance loops. The capacitive arc trend is the same across all tests, initially increasing, then decreasing, and ultimately turning negative. A higher impedance modulus |Z| in the low-frequency region indicates a stronger protective ability of the corresponding corrosion film.^29^ As soaking time increases, corrosion intensifies, predominantly manifesting as pitting corrosion, making it challenging to form a stable protective film on the sample surfaces. The impedance magnitude sequence for the four sample groups is T-1 (24 h) > T-2 (24 h) > T-1 (0 h) > T-2 (0 h). After initial exposure to soaking, more defects emerge on the sample surfaces, making them prone to pitting. The presence of such defects facilitates Cl^−^ aggregation, undermining the integrity of the surface film and accelerating corrosion. The differences in the impedance spectrum curves of T-1 and T-2 samples may stem from variations in corrosion rates, influencing the structure of the MgO/Mg(OH)_2_ oxide layer formed on the sample surfaces.

## Discussion

### Coupling test

To further investigate the micro-galvanic corrosion behavior of AZ31B magnesium alloy samples T-1 and T-2, both ZRA and SVET tests were conducted. The comprehensive results indicate significant micro-galvanic corrosion between the twin-zone T-2 sample and the matrix T-1 sample after coupling. Consistently, ZRA and SVET tests demonstrate that the T-2 sample, containing numerous {$10\bar{1}2$} tensile twins, acts as an anode accelerating corrosion, whereas the T-1 sample serves as a cathode, thus afforded protection. The ZRA test revealed a notable reduction in galvanic current between the T-1 and T-2 samples, with a critical turning point observed at 673 s. This suggests that at this juncture, micro-galvanic corrosion between the twin zone crystal planes and the matrix predominantly contributes to the increased dissolution rate of the alloy. In conjunction with the observations from Figure 8, the corrosion product film formed after soaking the AZ31B magnesium alloy in 37 ± 0.1°C simulated body fluid exhibits some protective effects against corrosion rate reduction. However, this protection is not predominant. Moreover, the SVET results displayed in Figure 12 indicate that the surface of the T-2 sample, containing {$10\bar{1}2$} tensile twins, shows pronounced corrosion behavior after 0 h, 1 h, and 2 h of soaking in simulated body fluid. This highlights the vulnerability of the T-2 sample to aggressive corrosion processes, particularly in regions with a high density of tensile twins.

### Corrosion resistance test

In the study of micro-galvanic corrosion of AZ31B magnesium alloy samples T-1 and T-2, measuring the galvanic current becomes challenging after 2 h due to the growth of the corrosion product film during the corrosion process. Electrochemical test results indicate that variations in twin density within the internal structure of T-1 and T-2 samples contribute to differences in electrochemical activity between the twin zones and the matrix. Initially, at 0 h, the corrosion current density of the T-1 sample is significantly lower than that of the T-2 sample, as shown in Figure 13A and Table 3. The sequence of corrosion current density across the samples is T-2 (0 h) > T-1 (0 h) > T-2 (24 h) > T-1 (24 h). Corrosion current, a kinetic parameter, reflects the corrosion rate; higher values indicate faster corrosion rates and a greater propensity for the matrix to corrode. Thus, the corrosion tendency of the AZ31B magnesium alloy samples is ranked as T-2 (0h) > T-1 (0h) > T-2 (24h) > T-1 (24 h). The appearance of the capacitive arc in the high-frequency area of the Nyquist plots typically signifies an electrochemical process related to changes in the electrode surface state within the electrochemical system. The interaction of charged ions in the simulated body fluid with the magnesium alloy leads to product deposition on the electrode surface, potentially forming compounds such as Ca-P. The corrosion product film on the magnesium alloy, when soaked in simulated body fluid, is likely formed through the following reactions^30^:

(Equation 1) $$Mg\to {Mg}^{2+}+2e$$

(Equation 2) $$2H_{2}O+2e^{-}\to H_{2}+2{OH}^{-}$$

(Equation 3) $$Mg+2{OH}^{-}\to Mg{\left(OH\right)}_{2}$$

(Equation 4) $$Mg{\left(OH\right)}_{2}+{2Cl}^{-}\to {MgCl}_{2}+{2OH}^{-}$$

**Figure 14:**
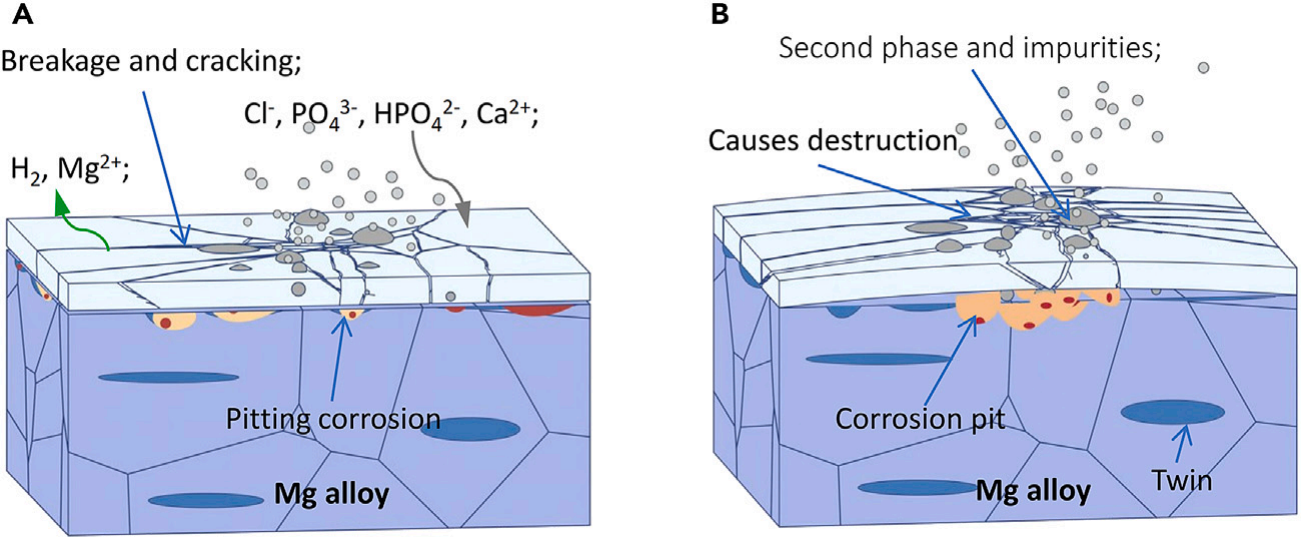
Schematic diagram of the corrosion mechanism of AZ31B magnesium alloy Initially, Mg(OH)_2_ deposits on the surface of the matrix, exhibiting loose and porous characteristics as depicted in Figure 14A. This allows Cl^−^ ions from the simulated body fluid to penetrate and dissolve these Mg(OH)_2_ deposits, resulting in the formation of soluble MgCl_2_. This process contributes to the continuous degradation of the Mg(OH)_2_ corrosion product film, as illustrated in Figure 14B.

In simulated body fluid, Mg undergoes hydrogen evolution galvanic corrosion, leading to the formation of Mg^2+^ ions. Initially, Mg(OH)_2_ deposits on the surface of the matrix, exhibiting loose and porous characteristics as depicted in Figure 14A. This allows Cl^−^ ions from the simulated body fluid to penetrate and dissolve these Mg(OH)_2_ deposits, resulting in the formation of soluble MgCl_2_. This process contributes to the continuous degradation of the Mg(OH)_2_ corrosion product film, as illustrated in Figure 14B. During the corrosion process, as described by the reaction in Equation 3, H_2_ and OH^−^ ions are produced. The OH^−^ ions react with Ca^2+^ and PO_4_^3−^ in the simulated body fluid to form Ca-P phase substances, represented by the reaction in Formula 6:

(Equation 5) $${Ca}^{2+}+{OH}^{-}+H_{2}{PO}_{4^{-}}+H_{2}O\to {CaHPO}_{4}2H_{2}O$$

This reaction explains why the bottom of the corrosion product film contains significant amounts of Mg(OH)_2_, whereas the top is enriched with Ca-P phase substances, a finding also verified in Figure 7. The appearance of the impedance arc in electrochemical testing is generally associated with disturbances in the alloy surface state, such as ion enrichment or bubble interference, indicating challenges in forming a stable protective film on the sample surface as soaking time increases.^31^ The size of the double-layer capacitance impedance arc is indicative of the charge transfer process at the interface. Initially, the T-2 sample exhibits the smallest impedance arc diameter at corrosion onset (0 h), which gradually increases over time, reaching the largest diameter at 24 h. Typically, a larger impedance arc in the Nyquist spectrum suggests better corrosion resistance. The Nyquist spectrum reveals a relatively large impedance arc size for the T-1 sample, indicating superior corrosion resistance.

As corrosion soaking time progresses, the emergence of a low-frequency impedance loop signifies localized corrosion on the sample surface. Over prolonged soaking, it becomes evident that the T-2 sample, which contains a significant number of {$10\bar{1}2$} tensile twins, is more reactive in 37 ± 0.1°C simulated body fluid compared to the T-1 sample. This enhanced reactivity leads to accelerated micro-galvanic corrosion between the twin zone and the matrix, thereby increasing the corrosion rate of the AZ31B magnesium alloy.

### Conclusions

In this study, uniform heat treatment and laser shock technology were employed to create two distinct microstructures within AZ31B magnesium alloy samples: the twin-free T-1 and T-2, which contains a significant number of {$10\bar{1}2$} tensile twins. Comparative analysis of these samples was conducted through SEM, SKPFM, ZRA testing, SVET testing, and comprehensive electrochemical assessments. The investigation focused on the influence of {$10\bar{1}2$} tensile twins on the corrosion behavior of AZ31B magnesium alloy in a simulated body fluid environment. The key findings are summarized as follows:

(1) SKPFM analysis revealed a notable Volta potential difference between the twin zone crystal planes and the matrix in the T-2 sample, with the twin zone exhibiting a more negative potential relative to the matrix. This indicates greater corrosion resistance in the matrix, a conclusion that is corroborated by the results displayed in Figure 4. The observed low potential difference also suggests the occurrence of micro-galvanic corrosion between the twins and the matrix.
(2) Coupling tests on AZ31B magnesium alloy samples demonstrated consistent results between ZRA and SVET evaluations for both T-1 and T-2 samples. Upon coupling, the T-2 sample, enriched with {$10\bar{1}2$} tensile twins, exhibited accelerated corrosion, acting as an anode, whereas the twin-free T-1 sample exhibited slower corrosion rates, acting as a cathode. The ZRA tests showed that the galvanic current for both T-1 and T-2 samples increased post-inflection point and remained positive, suggesting that the corrosion product film does not provide effective protection.
(3) Corrosion resistance testing indicated that the corrosion current for the T-1 sample was lower than that for the T-2 sample, with an increasing Nyquist radius. This finding confirms that the corrosion tendency of the AZ31B magnesium alloy samples when immersed in 37 ± 0.1°C simulated body fluid follows the order T-2 (0 h) > T-1 (0 h) > T-2 (24 h) > T-1 (24 h).

### Limitations of the study

The mechanism of {$10\bar{1}2$} twin in the degradation of AZ31B magnesium alloy has been investigated. Other types of magnesium alloys can also be used for reference and comparison. The degradation rate of AZ31B magnesium alloy in simulated body fluid is affected by multiple effects, and the twin in grain is only one of the factors. Since {$10\bar{1}2$} type twins have the highest content in AZ31B magnesium alloy matrix, only the response of {$10\bar{1}2$} type twins to corrosion is considered in the discussion of this problem. Whether this conclusion is also applicable to other types of magnesium alloys remains to be studied.

## STAR★Methods

### Key resources table

**Table:** 

REAGENT or RESOURCE	SOURCE	IDENTIFIER
**Chemicals, peptides, and recombinant proteins**		

simulated body fluid	Phygene	N/A

### Resource availability

#### Lead contact

Further information and requests for resources and information should be directed to and will be fulfilled by Xiangyu Li, (lixyu1995@163.com).

#### Materials availability

This study did not generate new unique materials.

#### Data and code availability

The datasets generated in this study is available on request by contacting Xiangyu Li, (lixyu1995@163.com).

### Experimental model and study participant details

No experimental models are used in this paper.

### Method details

#### Preparation and treatment of materials

The study utilizes as-cast AZ31B magnesium alloy samples to minimize the impact of original tissue texture on corrosion. The chemical composition of the samples conforms to the national standard GBT5153-2003, as detailed in Table 1. Each sample measures 10×10×5 mm^3^ and undergoes uniform heat treatment in a vacuum atmosphere tube furnace. The sample post heat treatment is labeled as T-1, whereas the sample subjected to both laser shock and uniform heat treatment is labeled as T-2. For T-1, the treatment temperature is set at 520°C for 4 hours, followed by cooling to room temperature within the furnace. The treatment parameters for T-2 include a laser power of 4.95 GW/cm^2^, two impacts, a 50 % spot overlap rate, and a subsequent annealing at 250°C for 1 hour to alleviate the residual stress induced by the laser shock. In this study, T-1 refers to the uniformly heat-treated sample without twins, and T-2 refers to the sample containing twins after laser shock.

#### Coupling test

To compare the corrosion behavior of AZ31B magnesium alloy samples T-1 and T-2, two distinct techniques, ZRA Test and SVET Test, are employed to assess the galvanic corrosion following coupling. Both tests are conducted in a 37±0.1°C simulated body fluid solution. The ZRA test utilizes the CHI600E electrochemical test system. For this test, T-1 and T-2 samples are each connected to a wire, encapsulated in epoxy resin to ensure fixation and sealing, with an exposed detection area polished to 10 mm^2^. The T-1 sample is connected to the ground of the electrochemical workstation, while the T-2 sample is attached to the electrode end. The workstation records the current every 5 seconds over a total duration of 2 hours.

The SVET test is performed using the VersaScan micro-area scanning electrochemical workstation. The T-1 and T-2 samples are coupled using conductive silver paste and connected via wires, followed by encapsulation in epoxy resin. The detection surface is polished to a mirror finish exposing an area of 200 mm^2^. The SVET tests are conducted over 1 and 2 hours in a 37±0.1°C simulated body fluid environment. During the test, the probe tip is maintained at a distance of 100 μm from the sample, utilizing a Pt-Ir microelectrode with a tip diameter of 16μm and an amplitude of 32 μm.

Coupling tests on AZ31B magnesium alloy samples demonstrated consistent results between ZRA and SVET evaluations for both T-1 and T-2 samples. Upon coupling, the T-2 sample, enriched with {} tensile twins, exhibited accelerated corrosion, acting as an anode, while the twin-free T-1 sample exhibited slower corrosion rates, acting as a cathode. The ZRA tests showed that the galvanic current for both T-1 and T-2 samples increased post-inflection point and remained positive, suggesting that the corrosion product film does not provide effective protection.

#### Electrochemical experiment

The PARSTAT electrochemical workstation is employed to perform electrochemical measurements in 37±0.1°C simulated body fluid. Measurements utilize a three-electrode sealed electrochemical cell, incorporating a saturated calomel electrode (SCE) as the reference electrode and a platinum electrode as the auxiliary electrode. The electrochemical corrosion sample is connected to a copper wire, encapsulated in epoxy resin, and the detection area, polished to 100 mm^2^, is exposed. The dynamic potential polarization curve test is conducted with a scanning speed of 1.0 mV/s. The anode and cathode scans are carried out separately to mitigate the effects of hydrogen evolution on the sample during corrosion. Electrochemical impedance spectroscopy is performed over a frequency range from 0.1 Hz to 100 kHz, with an excitation signal consisting of a sine wave of 5 mV amplitude, and data analysis is facilitated using ZsimpWin software. To ensure the stability of the electrolyte and to eliminate initial interference, a delay of 10 minutes is introduced before conducting the experiments. All electrochemical tests are replicated 2-3 times following the same procedure to confirm the reliability of the results.

Corrosion resistance testing indicated that the corrosion current for the T-1 sample was lower than that for the T-2 sample, with an increasing Nyquist radius. This finding confirms that the corrosion tendency of the AZ31B magnesium alloy samples when immersed in 37±0.1°C simulated body fluid follows the order: T-2 (0 h) > T-1 (0 h) > T-2 (24 h) > T-1 (24 h).
